# Artesunate Therapy Alleviates Fracture-Associated Chronic Pain After Orthopedic Surgery by Suppressing CCL21-Dependent TREM2/DAP12 Inflammatory Signaling in Mice

**DOI:** 10.3389/fphar.2022.894963

**Published:** 2022-06-02

**Authors:** Linlin Zhang, Nan Li, Haoyue Zhang, Yigang Wang, Tianyu Gao, Yuying Zhao, Guolin Wang, Yonghao Yu, Chunyan Wang, Yize Li

**Affiliations:** ^1^ Department of Anesthesiology, Tianjin Medical University General Hospital, Tianjin, China; ^2^ Tianjin Research Institute of Anesthesiology, Tianjin, China

**Keywords:** artesunate, bone fracture, CCL21, chronic pain, DAP12, TREM2, spinal cord

## Abstract

Chronic pain after bone fracture and orthopedic surgery is often refractory to most analgesics currently in use, thus emphasizing the urgent need for improved therapeutic medications. Chemokine-dependent neuroinflammation is critical for excitatory synaptic plasticity and central nociception sensitization. Recent studies have focused on the inhibition of inflammatory responses by artesunate, the first anti-malaria drug extracted from artemisinin. The present study investigated the analgesic effects and potential targets of artesunate in a mouse model of chronic pain induced by tibial fracture and orthopedic surgery. Three injections of artesunate were intrathecally administered on a daily basis from days 4 to 6 after fracture. We reported that repetitive exposure to artesunate (10 and 100 μg but not 1 μg) dose-dependently prevented fracture-induced mechanical and cold allodynia. Moreover, single intrathecal injection of artesunate (100 μg) alleviated the established chronic pain on day 14 after fracture surgery. Intraperitoneal artesunate (10 and 50 mg kg^−1^) therapy was effective against chronic fracture pain. Intriguingly, artesunate inhibited the upregulation of spinal chemokine CCL21, triggering receptor expressed on myeloid cells 2 (TREM2) and DNAX-activating protein of 12 kDa (DAP12) expressions and microglia activation in fracture mice. Furthermore, spinal CCL21 neutralization attenuated the severity of fracture-associated post-surgical pain. Exogenous CCL21-induced acute inflammatory pain was impaired by artesunate therapy. Additionally, the pharmacological blockage of TREM2 reduced recombinant CCL21-elicited behavioral hypernociception. The present findings demonstrate that artesunate therapy reduces the initiation and maintenance of fracture-associated chronic postoperative pain by inhibiting CCL21-dependent TREM2/DAP12 inflammatory signaling and microglia activation, thus suggesting that artesunate could emerge as a therapeutic strategy for fracture pain management.

## Introduction

With the rapid development of industry, construction, and transportation and the aggravation of population aging, the occurrence of fracture following industrial accidents, construction injuries, traffic injuries, and osteoporosis in the elderly also increases (Chen et al., 2017). Chronic pain after bone fracture and orthopedic surgery is clinically common and imposes a heavy financial burden to patients worldwide. It has been reported that the incidence of chronic pain after ankle and wrist fractures is 61.7% and that the incidence of chronic pain after tibial fracture is 55.1% (Friesgaard et al., 2016; Khan et al., 2016). Treatment of fracture pain remains a dramatic challenge to pain physicians. Recent reports have recapitulated the requirement of neuroinflammation for spinal pain sensitization, which is critical for multiple nociceptive perceptions after peripheral nerve injury, cancer, and bone fracture (Chen et al., 2018; Baral et al., 2019; Li et al., 2021). Yet, the specific molecular pathogenesis underlying fracture pain continues to be elusive.

Chemokine-mediated neuroinflammation involves microglia activation and neuronal plasticity in pain neurocircuits that, subsequently, maintain the pain phenotype (Ji et al., 2014; Ji et al., 2016). Chemokine CCL21, as a cardinal microglia-activating factor localized in dorsal horn neurons, is indicated to mediate excitatory synaptic transmission via microgliosis in pain states, ranging from chronic neuropathic pain to persistent cancer pain (Biber et al., 2011; Zheng et al., 2019; Hirth et al., 2020). Triggering receptors expressed on myeloid cells 2 (TREM2) and DNAX-activating protein of 12 kDa (DAP12) in microglia are gradually recognized as the downstream of microglial inflammatory signaling in pronociceptive facilitation during chemotherapy-induced peripheral neuropathy and nerve trauma-induced neuropathic allodynia (Hu et al., 2018; Wang Y. et al., 2020). Nevertheless, whether spinal CCL21 contributes to fracture-associated chronic pain via TREM2/DAP12 pathway remains largely unknown.

Given that current analgesics including opioid agents and non-steroidal anti-inflammatory drugs have several side effects and may interfere with bone healing (Kidner et al., 2009; Lisowska et al., 2018; Tracey et al., 2019), alternative agents for fracture-associated chronic pain control are urgently required. Artesunate, as an active derivative of artemisinin (Qinghaosu) with little toxicity, has been generally utilized to treat malaria for recent decades (Zou et al., 2020). Remarkably, artemisinin and its derivatives provide multi-therapeutic protections of anti-inflammation, anti-oxidation, anti-cancer, and anti-viral infection in a wide variety of pathophysiological disorders, such as sepsis, neurodegeneration, ischemia-reperfusion injury, tumors, and severe coronavirus disease (COVID-19) caused by SARS-CoV-2 (Duan et al., 2019; Gendrot et al., 2020; Kasaragod et al., 2020; Bang et al., 2021). Furthermore, artesunate is recently identified as an effective analgesic prescription for remifentanil-induced hyperalgesia and complete Freund’s adjuvant (CFA)-induced acute inflammatory pain via the maintenance of oxidative homeostasis in rodents (Guruprasad et al., 2015; Zhang et al., 2022). However, whether artesunate ameliorates fracture-associated chronic pain by modulating spinal inflammatory responses requires to be further investigated.

In this study, we characterized the potential role of intrathecal (i.t.) artesunate in chronic postoperative pain using a mouse model of tibial fracture with intramedullary pinning. Spinal expressions of CCL21, TREM2, and DAP12 were measured to verify the nociceptive pathogenesis and anti-nociceptive targets of artesunate in our orthopedic model. Our findings identified that the inhibition of neuroinflammation by artesunate may offer a novel therapeutic strategy for pain control after orthopedic surgery.

## Materials and Methods

### Animals

Adult male C57BL/6J mice, 8–10 weeks old, were raised in an artificially regulated 12-h light–dark environment with food and water *ad libitum*. All animals were purchased from the experimental animal center of the Chinese Academy of Military Medical Science. All experimental studies and protocols were conducted in strict accordance with the International Association for the Study of Pain directives and approved by the Animal Ethical and Welfare Committee of Tianjin Medical University (Tianjin, China).

### Drugs and Administration

Artesunate (MedChemExpress, HY-N0193, China) was dissolved in 10% dimethyl sulfoxide (DMSO, Sigma-Aldrich, D2650, United States) for i.t. injection. Recombinant CCL21 (Abcam, ab201361, United Kingdom), a neutralizing antibody against CCL21 (anti-CCL21, R&D Systems, AF457, United States) and a neutralizing antibody against TREM2 (anti-TREM2, R&D Systems, 1729-T2, United States), was dissolved in normal saline or 10% DMSO for i.t.; delivery and the doses of these reagents were chosen based on previous reports (Hu et al., 2018; Wang et al., 2020a; Zhang et al., 2022). The intrathecal injection was performed under brief anesthesia of sevoflurane (induction, 3.0%; surgery, 1.5%; Maruishi Pharmaceutical Co., Ltd., Japan) and made between the levels of L_5_ and L_6_ using a 30-G needle (Donnelly et al., 2021); 5 μl of the reagent was given when the reflexive tail flick was observed.

### Surgery

The tibial fracture-associated postoperative pain model was established according to the previously described procedures (Zhang et al., 2018; Cui et al., 2021). Briefly, animals were anesthetized with sevoflurane (induction, 3.0%; surgery, 1.5%) by a nose mask under sterile conditions. Muscles were disassociated following an incision from the knee to the midshaft of the left tibia. After osteotomy, a 0.38-mm stainless steel pin was inserted into the tibia intramedullary canal, and the incision was sutured with 6–0 prolene. For sham operation, the incision and muscle disassociation were made identically without tibial fracture with pinning.

### Behavioral Testing

All tests were conducted between 10:00 a.m. and 3:00 p.m. on that day in a temperature-controlled room at 24°C. The baseline threshold was tested 1 day before the treatment, and the mice were habituated 2 h per day in the testing apparatus for 3 days prior to the baseline threshold test.

In the von Frey test, the mice were placed on a test platform with a grid spacing of 1.5 mm, covered with a plexiglass box of 49 cm × 33 cm × 40 cm, and allowed to acclimatize for 2 h. The paw withdrawal threshold (PWT) of the mice was measured with the von Frey filaments (Stoelting, United States) between 0.16 and 2 g using the up-and-down method. Starting with 0.16 g to stimulate the left hind paw, licking or withdrawal during the 5 s stimulus was considered as a positive response. The force was reduced in the case of a positive reaction; otherwise, the force was increased, and finally the PWT was calculated (Zhang et al., 2018; Cui et al., 2021). With reference to the frequency behaviors, a 0.16 g von Frey filament was used to stimulate left hind paws for 10 times with a 30 s interval, and the percentage of withdrawal responses was calculated as frequency (Zhang et al., 2018; Zhang et al., 2021).

For measurement of cold allodynia, two acetone applications (20 μl each) were gently applied to the left hind paw bottom using a pipette and the responses to acetone were scored: 0, no response; 1, quick withdrawal, paw stamping, or flicking; 2, prolonged withdrawal or repeated flicking of the paw; and 3, repeated paw flicking and licking (Cui et al., 2021; Zhang et al., 2021).

The same investigator blinded to the treatments collected the behavioral data.

### ELISA Analysis

An enzyme-linked immunosorbent assay (ELISA) was used to measure the concentrations of CCL21 (ab208985, Abcam), TREM2 (SAB2501170, Sigma), and DAP12 (EM8531, Wuhan Fine Biotech Co., China) in the L_4-5_ segments of spinal cord. The spinal cord tissues were collected before and after tibial fracture, as well as at hour 12 after exogenous CCL21 administration. The spinal cord tissues were homogenized in a lysis buffer containing protease and phosphatase inhibitors. The tissue samples were centrifuged at 12,500 ×g for 10 min, and the supernatant was collected. The BCA protein assay (Pierce) was employed to determine protein concentrations. For each reaction in a 96-well plate, 100 μg of proteins of samples were used. All ELISA experiments followed the manufacturer’s protocol. The optical densities of samples were measured using an ELISA plate reader (Bio-Rad) at a wavelength of 450 nm, and the levels of CCL21, TREM2, and DAP12 were calculated using the standard curves and normalized to the total protein levels.

### Immunofluorescence

The mice were deeply anesthetized and transcardially perfused with pre-cooled PBS following 4% paraformaldehyde. The whole spinal cord was blown out using the hydraulic pressure method. The L_4_–L_5_ spinal cord was dissected and dehydrated in 30% sucrose for 2 days. The tissues were then frozen in OCT and cut into 5-µm frozen sections using a cryostat (Leica Biosystems, Germany). The sections were blocked with 0.3% Triton X-100 for 10 min and 5% goat serum for 1 h. They were then incubated with primary antibodies overnight at 4°C. The following primary antibody was used: anti-Iba-1 (1:200, Abcam, ab178847, United Kingdom). After rinsing three times with PBS, the sections were incubated with a fluorescence-labeled secondary antibody for 1 h. Images were collected using a fluorescence microscope (Olympus, Japan), and the analysis was performed using Image J software.

### Statistical Analysis

All statistical analyses were performed with SPSS 18.0 software (SPSS, United States). All animals were randomly assigned to experimental conditions. All data were expressed as mean ± standard error of mean (SEM). The sample size was calculated as previously described (Zhang et al., 2018; Cui et al., 2021; Zhang et al., 2021). The Shapiro–Wilk test was used for determining the normality of data distribution, and parametric statistics were applied. The homogeneity of variance was validated using the Levene test. The statistical analyses of behavioral data were performed by two-way analysis of variance (ANOVA) with Bonferroni *post hoc* comparisons. The results of biochemical experiments were analyzed using one-way ANOVA with Bonferroni *post hoc* comparisons. A *p* value <0.05 was considered statistically significant.

## Results

### Tibial Fracture Generates Chronic Postoperative Pain and Increases Spinal Expressions of CCL21, TREM2, and DAP12 After Orthopedic Surgery

**FIGURE 1:**
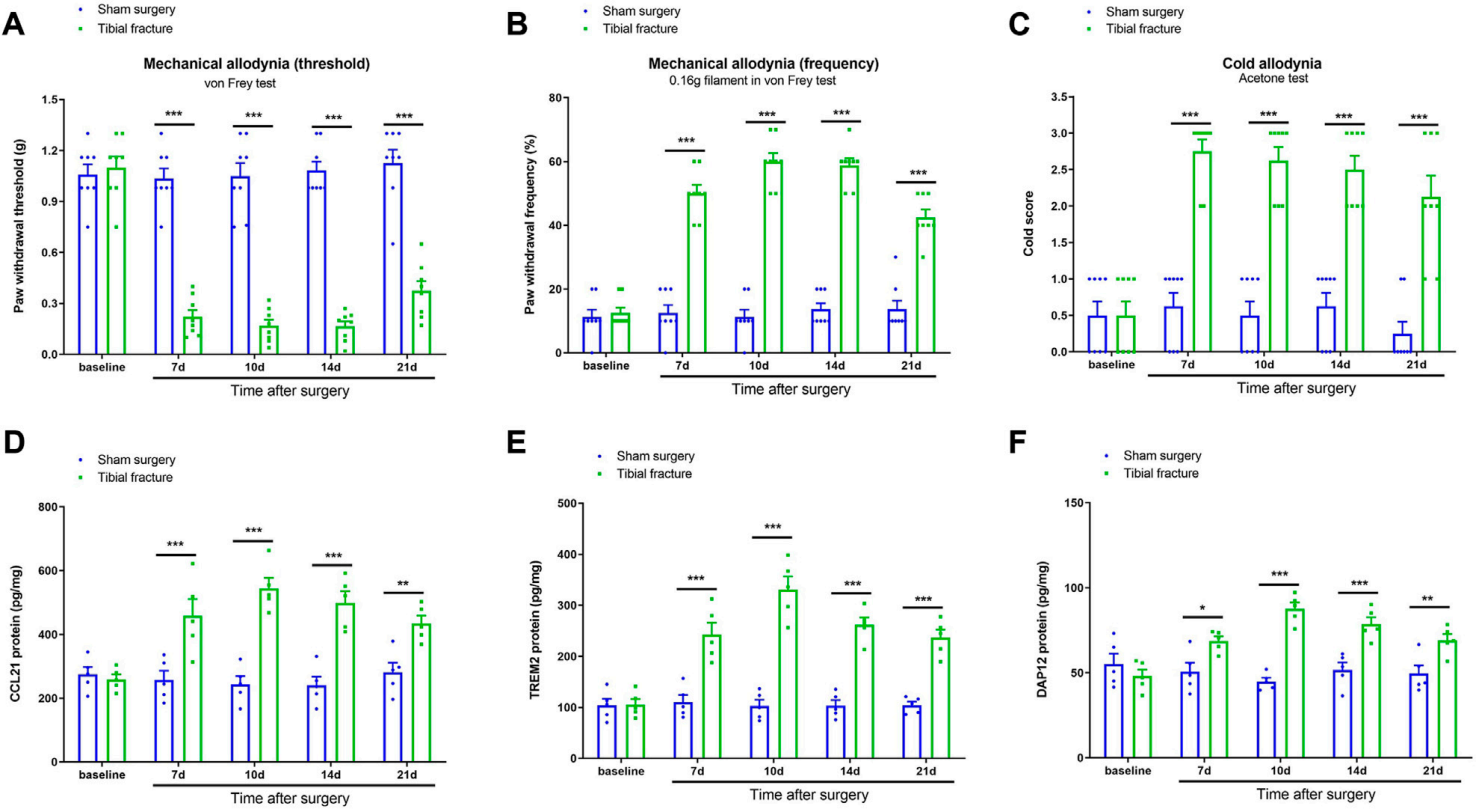
Fracture-associated behavioral allodynia and spinal over-expressions of CCL21, TREM2, and DAP12 after orthopedic surgery in mice. The development of mechanical allodynia was assessed by paw withdrawal mechanical threshold **(A)** and paw withdrawal mechanical frequency to 0.16 g filament **(B)** in the von Frey test after sham surgery and tibial fracture (*n* = 8). The development of cold allodynia was assessed by cold response scoring **(C)** in acetone test after sham surgery and tibial fracture (*n* = 8). The spinal dorsal horn L_4_-_5_ segments were collected for biochemical experiments. **(D–F)** ELISA identified the increased levels of spinal CCL21, TREM2, and DAP12 proteins after tibial fracture (*n* = 5). All the data are expressed as mean ± SEM and analyzed by two-way ANOVA with Bonferroni *post hoc* comparisons. *p < 0.05, **p < 0.01, ***p < 0.001 vs. group sham surgery.

First, we did not observe any significant differences in basal mechanical and cold sensitivities between two groups (*p* > 0.05, *n* = 8; Figures 1A–C). The von Frey test revealed that sham surgery did not change the postoperative paw withdrawal threshold and frequency in comparison with baseline (*p* > 0.05, *n* = 8; Figures 1A,B). The acetone test did not detect any marked alternation in cold response scores after sham operation (*p* > 0.05, *n* = 8; Figure 1C). Interestingly, as compared to sham animals, tibial fracture caused persistent (>21 days) postoperative pain (mechanical allodynia), as indicated by significantly decreased paw withdrawal thresholds [F (1, 70) = 332.5, *p* < 0.001, *n* = 8, two-way ANOVA; Figure 1A] and increased paw withdrawal frequency [F (1, 70) = 471.6, *p* < 0.001, *n* = 8, two-way ANOVA; Figure 1B] after orthopedic treatment. As parallel, the postoperative pain represented prolonged cold allodynia by a long-lasting elevation of cold response following fracture with pin insertion, as compared to sham intervention [F (1, 70) = 166.7, *p* < 0.001, *n* = 8, two-way ANOVA; Figure 1C].

Neuroinflammation-driven synaptic plasticity in the spinal cord dorsal horn is a central feature of pathological pain with different etiologies (Ji et al., 2014; Ji et al., 2018; Qiang and Yu, 2019; Wang et al., 2020b). Thus, the present study emphasized the specific molecular signaling of spinal nociceptive information transmission following fracture with pin insertion. ELISA exhibited that sham operation failed to affect the expression of CCL21, TREM2, and DAP12 in the spinal dorsal horn (*p* > 0.05, *n* = 5; Figures 1D–F). Noteworthy, our biochemical results showed that spinal levels of CCL21, TREM2, and DAP12 were considerably upregulated within 7 days, peaked at 14 days, and continued for at least 21 days in animals undergoing tibial fracture and orthopedic surgery (*p* < 0.05, *n* = 5; Figures 1D–F), which was consistent with the time course of chronic postoperative pain phenotypes. All these data suggest that tibial fracture with pin insertion initiates persistent spinal over-expression of CCL21, TREM2, and DAP12, which may be essential for the pathogenesis of chronic fracture pain.

### Intrathecal Pretreatment With Artesunate Prevents Persistent Postoperative Pain Following Tibial Fracture and Orthopedic Surgery

**FIGURE 2:**
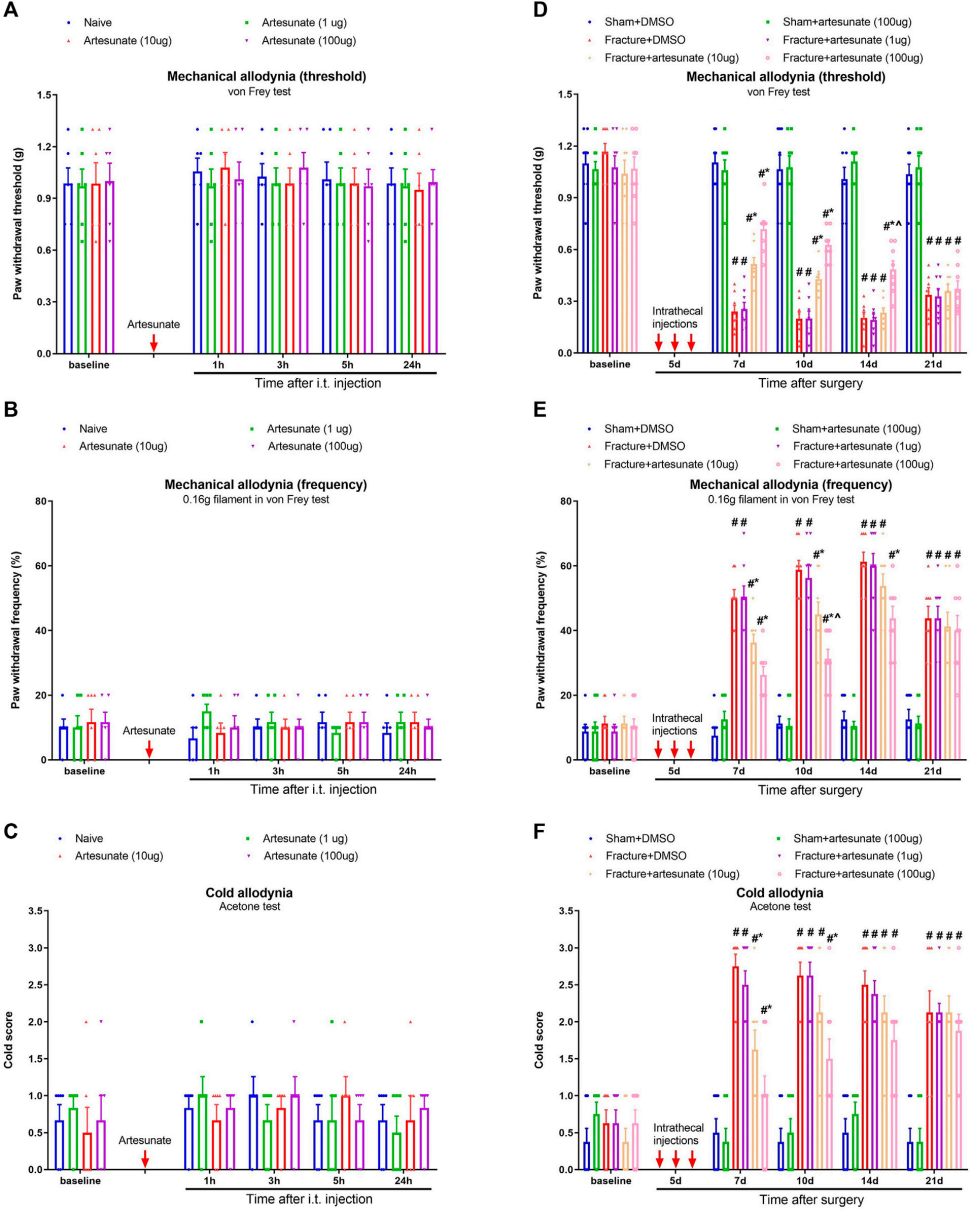
Intrathecal pre-administration of artesunate reduces fracture-associated postoperative pain. **(A–C)** Single injection of artesunate (1, 10, and 100 μg) was intrathecally administered in naïve mice. **(D–F)** Intrathecal artesunate (1, 10, and 100 μg) was administered daily for three consecutive days on days 4, 5, and 6 (indicated by red arrows) after tibial fracture with orthopedic surgery. Also, artesunate (i.t., 100 μg) was injected on days 4, 5, and 6 after sham surgery. All behavioral results are mean ± SEM (*n* = 6–8) and analyzed by two-way ANOVA with Bonferroni *post hoc* comparisons. ^#^ *p* < 0.05 vs. group sham + DMSO, *p < 0.05 vs*.* group fracture + DMSO, ^^^ *p* < 0.05 vs*.* group fracture + artesunate (10 μg).

To examine the effect of artesunate on basal nociceptive sensitivity, i.t., artesunate (1, 10, and 100 μg) was injected in naïve animals. We found that as compared to baseline, artesunate treatment did not impair peripheral mechanical and cold sensitivity in naïve mice (*p* > 0.05, *n* = 6; Figures 2A–C), suggesting that DHA therapy at 1, 10, and 100 μg was safe for our model. Then, to explore the potential role of artesunate in chronic fracture pain, i.t., artesunate (1, 10, and 100 μg) was administered daily for three consecutive days on days 4–6 (in the early phase) after tibial fracture with orthopedic surgery. Also, artesunate (i.t., 100 μg) was injected on days 4–6 in animals after sham surgery. Intriguingly, the i.t. therapy of artesunate at 10 and 100 μg but not 1 μg reduced fracture-associated postoperative pain, as characterized by the abrupt increase in paw withdrawal mechanical threshold [F (5, 210) = 173.8, *p* < 0.001, *n* = 8, two-way ANOVA; Figure 2D], the significant decrease in paw withdrawal mechanical frequency [F (5, 210) = 127.0, *p* < 0.001, *n* = 8, two-way ANOVA; Figure 2E], and the considerable reduction in cold scores [F (5, 210) = 64.93, *p* < 0.001, *n* = 8, two-way ANOVA; Figure 2F]. Such analgesia of artesunate strongly lasted for 1–7 days after the termination of the third treatment in a dose-dependent manner.

**FIGURE 3:**
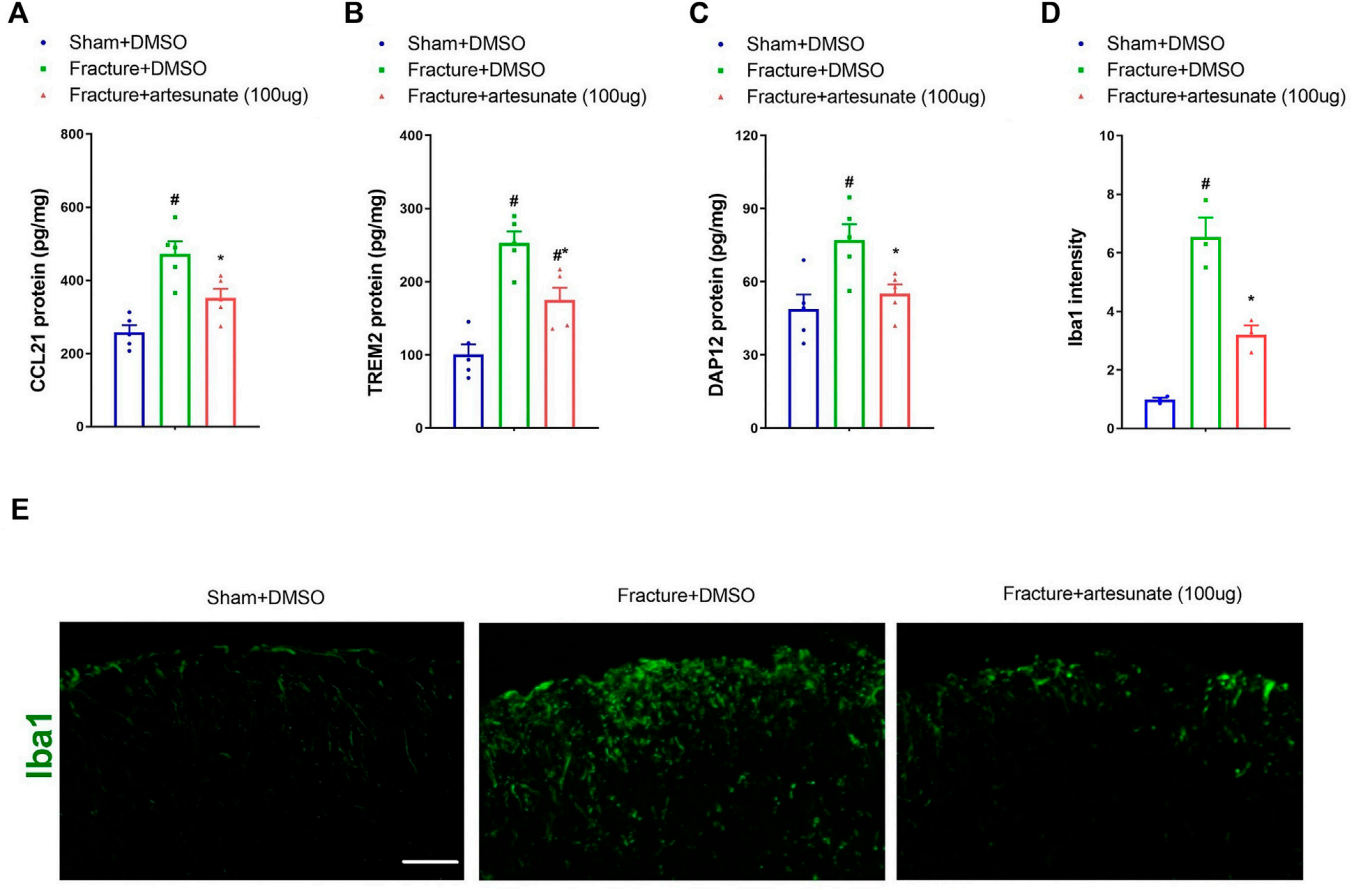
Intrathecal pre-administration of artesunate reduces fracture-associated spinal over-expressions of CCL21, TREM2, and DAP12 and microglia activation. Intrathecal artesunate (100 μg) was administered daily for three consecutive days on days 4, 5, and 6 after tibial fracture with orthopedic surgery. All biochemical data were collected on day 7 after sham and fracture surgeries. **(A–C)** ELISA identified that pretreatment with artesunate downregulated the increased levels of spinal CCL21, TREM2, and DAP12 proteins after tibial fracture. **(D,E)** Immunohistochemistry staining showed representative photomicrographs of the marker of microglia activation (Iba1) in the spinal dorsal horn after fracture intervention and artesunate treatment (scale bar, 50 μm). All biochemical results are expressed as mean ± SEM (*n* = 3–5) and analyzed by one-way ANOVA with Bonferroni *post hoc* comparisons. ^#^ *p* < 0.05 vs. group sham + DMSO, *p < 0.05 vs. group fracture + DMSO.

We further detected the levels of CCL21 and TREM2/DAP12 to verify whether these inflammatory mediators were involved in artesunate analgesia in fracture mice. Notably, artesunate pretreatment (100 μg) reduced the spinal up-modulation of CCL21, TREM2, and DAP12 expressions after tibial fracture with pin insertion (*p* < 0.05, *n* = 5; Figures 3A–C). Microglia activation in the spinal dorsal horn of mice with fracture pain has been revealed in previous reports (Li et al., 2015; Shi et al., 2015; Zhang et al., 2016). Herein, we also found the suppression of fracture-related spinal microglia activation by artesunate treatment (*p* < 0.05, *n* = 3; Figures 3D,E). All these data suggest that artesunate therapy prevents chronic fracture pain *via* spinal inhibition of neuroinflammation.

### Postoperative Treatment With Intrathecal Artesunate Impaired the Established Persistent Pain After Tibial Fracture and Orthopedic Surgery

**FIGURE 4:**
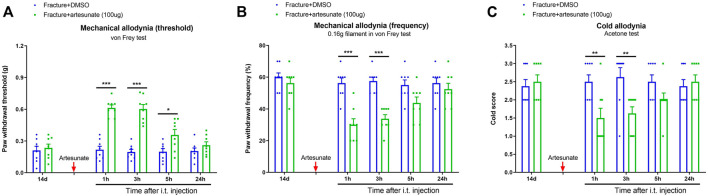
Intrathecal post-treatment of artesunate impairs the established chronic pain after tibial fracture and orthopedic surgery. Artesunate (i.t., 100 μg, indicated by a red arrow) was injected on day 14 (in the late phase) after tibial fracture with pin insertion. The development of mechanical allodynia was assessed by paw withdrawal mechanical threshold **(A)** and paw withdrawal mechanical frequency to 0.16 g filament **(B)** in the von Frey test after tibial fracture. The development of cold allodynia was assessed by cold response scoring **(C)** in acetone test after tibial fracture. Results are mean ± SEM (*n* = 8) and analyzed by two-way ANOVA with Bonferroni *post hoc* comparisons. *p < 0.05, **p < 0.01, ***p < 0.001 vs. group fracture + DMSO.

After the prevention of fracture pain by artesunate was conformed, we further assessed the efficiency of postoperative i.t., artesunate therapy at improving chronic pain. The von Frey tests detected that the single administration of artesunate (i.t., 100 μg) on 14 days after fracture (in the late phase) produced a rapid and transient attenuation of the established mechanical allodynia for 5 h, as reflected by the increase in paw withdrawal threshold [F (1, 70) = 86.87, *p* < 0.001, *n* = 8, two-way ANOVA; Figure 4A] and the decrease in paw withdrawal frequency [F (1, 70) = 45.34, *p* < 0.001, *n* = 8, two-way ANOVA; Figure 4B] in fracture animals. As parallel, this therapy of artesunate also suppressed the established cold allodynia for 3 h [F (1, 70) = 12.06, *p* < 0.001, *n* = 8, two-way ANOVA; Figure 4C].

### Spinal Inhibition of CCL21 Attenuates Tibial Fracture-Associated Postoperative Pain

**FIGURE 5:**
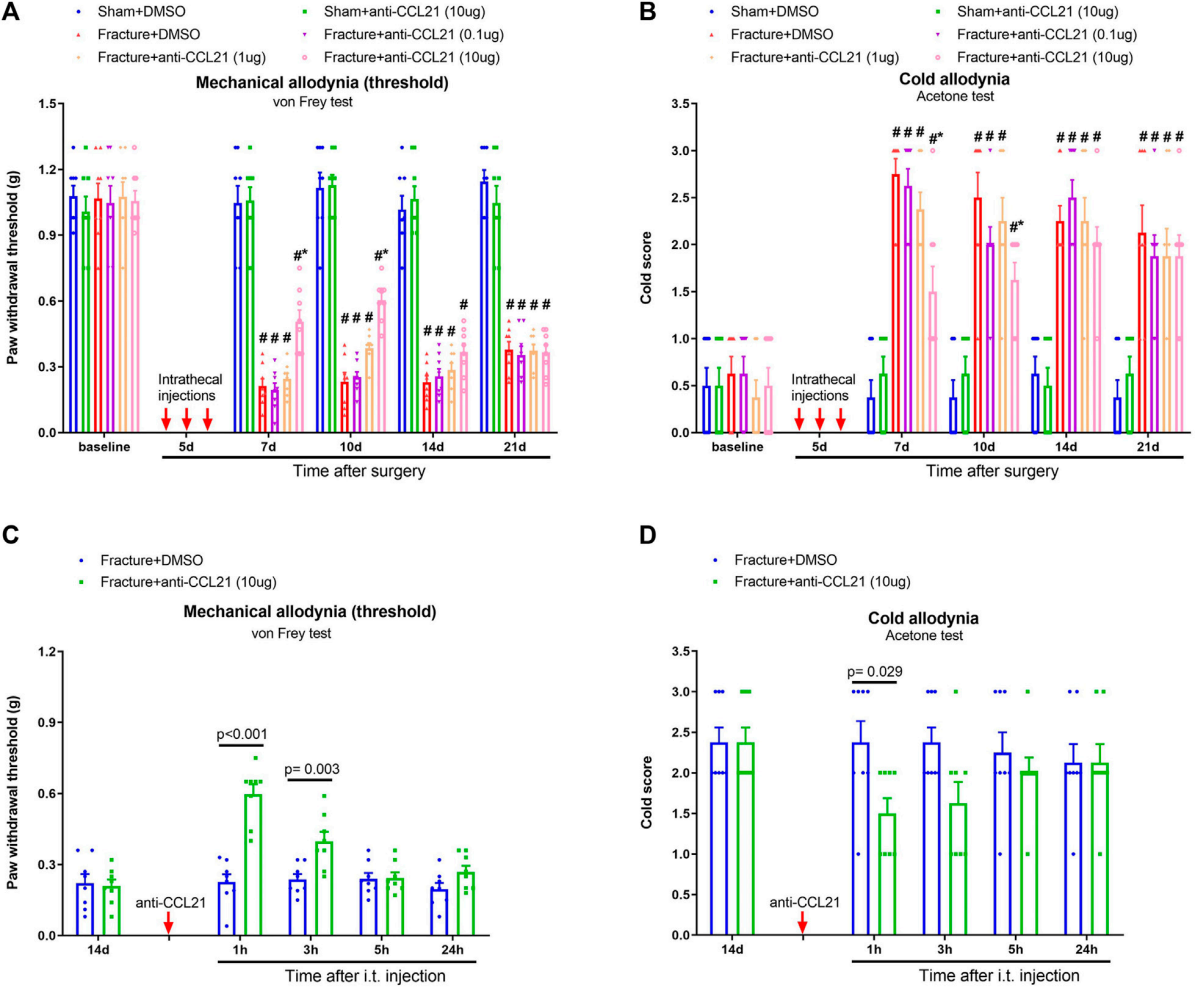
Spinal neutralization of CCL21 reduces fracture-associated postoperative pain. **(A,B)** A neutralizing antibody against CCL21 (anti-CCL21) was intrathecally injected (0.1, 1 and 10 μg) on days 4, 5, and 6 (indicated by red arrows) after fracture surgery. Mechanical allodynia and cold allodynia were measured by the von Frey test and acetone test, respectively. **(C,D)** Intrathecal injection of anti-CCL21 (10 μg) on day 14 (indicated by a red arrow) after fracture surgery significantly inhibited the established mechanical and cold allodynia. All behavioral results are mean ± SEM (*n* = 8) and analyzed by two-way ANOVA with Bonferroni *post hoc* comparisons. ^#^ *p* < 0.05 vs. group sham + DMSO, *p < 0.05 vs. group fracture + DMSO.

To further examine whether the CCL21 pathway is central for the development of chronic fracture pain, repetitive anti-CCL21 (i.t., 0.1, 1, and 10 μg) was delivered on a daily basis from days 4 to 6 (in the early phase) after tibial fracture with pin insertion. Strikingly, pretreatment with anti-CCL21 at 10 μg but not 0.1 and 1 μg generated a significant alleviation of mechanical and cold allodynia, which sustained for more than 4 days after three injections, as demonstrated by the increase of paw withdrawal threshold [F (5, 210) = 192.9, *p* < 0.001; *n* = 8, two-way ANOVA; Figure 5A] and the decrease of cold scores [F (5, 210) = 57.76, *p* < 0.001, *n* = 8, two-way ANOVA; Figure 5B] in fracture-treated animals. Moreover, the single injection of anti-CCL21 (10 μg) on day 14 after fracture intervention attenuated the established mechanical allodynia for 3 h [F (1, 70) = 36.96, *p* < 0.001, *n* = 8, two-way ANOVA; Figure 5C] and cold allodynia for 1 h [F (1, 70) = 7.39, *p* = 0.008, *n* = 8, two-way ANOVA; Figure 5D]. Thus, these behavioral data suggested that the spinal CCL21 pathway contributes to the production and persistence of chronic postoperative pain following tibial fracture procedures.

### Intrathecal Artesunate Therapy Protects Against CCL21-Induced Acute Pain Phenotype and Spinal Increases of TREM2 and DAP12

**FIGURE 6:**
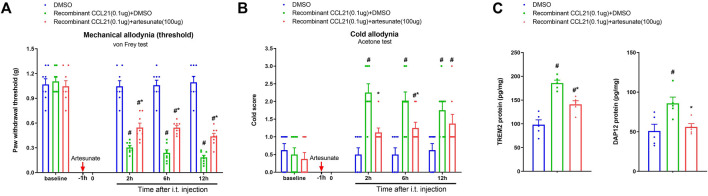
Induction of acute pain by exogenous CCL21 and attenuation of allodynia by artesunate. Artesunate (i.t., 100 μg, indicated by a red arrow) was injected 60 min prior to recombinant CCL21 (i.t., 0.1 μg) application. **(A,B)** Exogenous CCL21-induced acute inflammatory pain was alleviated by the pre-application of artesunate. All behavioral results are mean ± SEM (*n* = 8) and analyzed by two-way ANOVA with Bonferroni *post hoc* comparisons. All biochemical data were collected at hour 12 after intrathecal injection. **(C)** ELISA identified that pretreatment with artesunate downregulated the increased levels of spinal TREM2 and DAP12 proteins after exogenous CCL21 exposure. All biochemical results are expressed as mean ± SEM (*n* = 5) and analyzed by one-way ANOVA with Bonferroni *post hoc* comparisons. ^#^ *p* < 0.05 vs. group DMSO, *p < 0.05 vs. group recombinant CCL21 (0.1 μg) + DMSO.

Then, we further tested the hypothesis that artesunate would control the spinal CCL21-dependent nociception sensitization. We previously reported that acute exposure to recombinant CCL21 (i.t., 0.1 μg) directly initiated an acute allodynia (Wang et al., 2020a). Herein, artesunate (i.t., 100 μg) was injected 60 min prior to CCL21 administration. Interestingly, we found that artesunate intervention ameliorated CCL21-associated mechanical allodynia [F (2, 84) = 142.1, *p* < 0.001, *n* = 8, two-way ANOVA; Figure 6A] and cold allodynia [F (2, 84) = 26.39, *p* < 0.001, *n* = 8, two-way ANOVA; Figure 6B]. Furthermore, intrathecal exposure to CCL21 elevated the spinal concentrations of TREM2 and DAP12, which was compromised by artesunate pretreatment (*p* < 0.05, *n* = 5; Figure 6C). Collectively, these detailed data further illustrated that CCL21-dependent inflammatory signaling (TREM2/DAP12) might be a therapeutic target of artesunate analgesia in pain conditions.

### Pharmacological Inhibition of TREM2 Reduces Fracture-Associated Chronic Pain and CCL21-Induced Acute Pain

**FIGURE 7:**
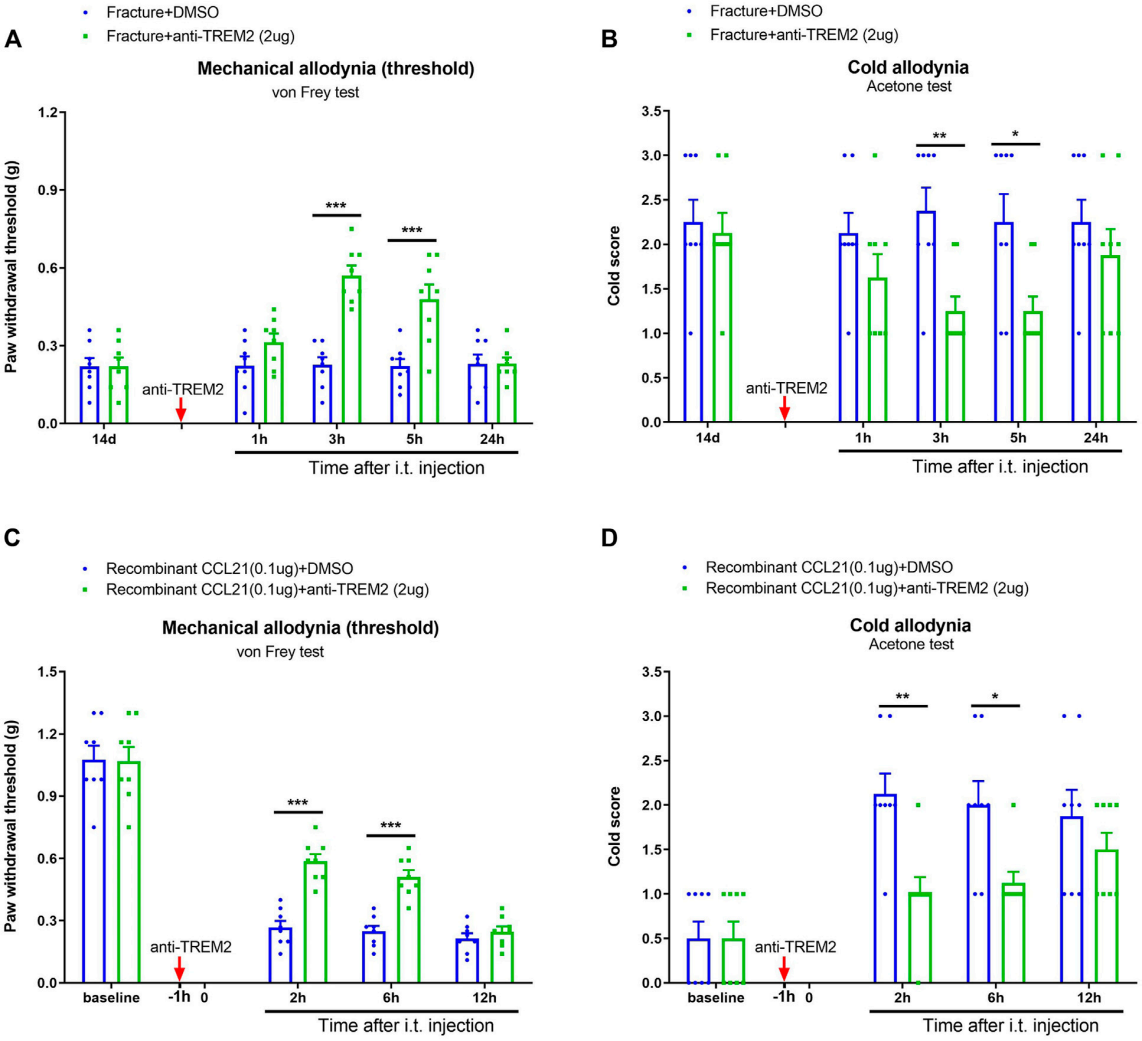
Spinal neutralization of TREM2 reduces fracture-associated chronic pain and CCL21-induced acute pain. **(A,B)** A neutralizing antibody against TREM2 (anti-TREM2, i.t., 2 μg, indicated by a red arrow) was injected on day 14 after tibial fracture. Behavioral test showed the attenuation of the established fracture-associated mechanical allodynia and cold allodynia by anti-TREM2. **(C,D)** Anti-TREM2 (i.t., 2 μg, indicated by a red arrow) was injected 60 min prior to recombinant CCL21 (i.t., 0.1 μg). Exogenous CCL21-induced acute inflammatory pain was alleviated by the pre-application of anti-TREM2. All results are mean ± SEM (*n* = 8) and analyzed by two-way ANOVA with Bonferroni *post hoc* comparisons. *p < 0.05, **p < 0.01, ***p < 0.001.

Next, we evaluated the effect of TREM2 signaling on pathological pain development. Intriguingly, a neutralizing antibody against TREM2 (anti-TREM2, i.t., 2 μg) ameliorated the established mechanical allodynia [F (1, 70) = 37.75, *p* < 0.001, *n* = 8, two-way ANOVA; Figure 7A] and cold allodynia [F (1, 70) = 16.14, *p* < 0.001, *n* = 8, two-way ANOVA; Figure 7B following tibial fracture with pin insertion. As parallel, the pre-administration of anti-TREM2 (i.t., 2 μg) reduced the exogenous CCL21-induced upregulation of peripheral mechanical sensitivity [F (1, 56) = 25.2, *p* < 0.001, *n* = 8, two-way ANOVA; Figure 7C] and cold sensitivity [F (1, 56) = 15.32, *p* < 0.001, *n* = 8, two-way ANOVA; Figure 7D]. These results indicate a substantial interaction between CCL21 and TREM2/DAP12 in spinal neuroinflammation and pain transduction.

### Systemic Therapy of Artesunate Alleviates Fracture-Associated Behavioral Pain After Orthopedic Surgery

**FIGURE 8:**
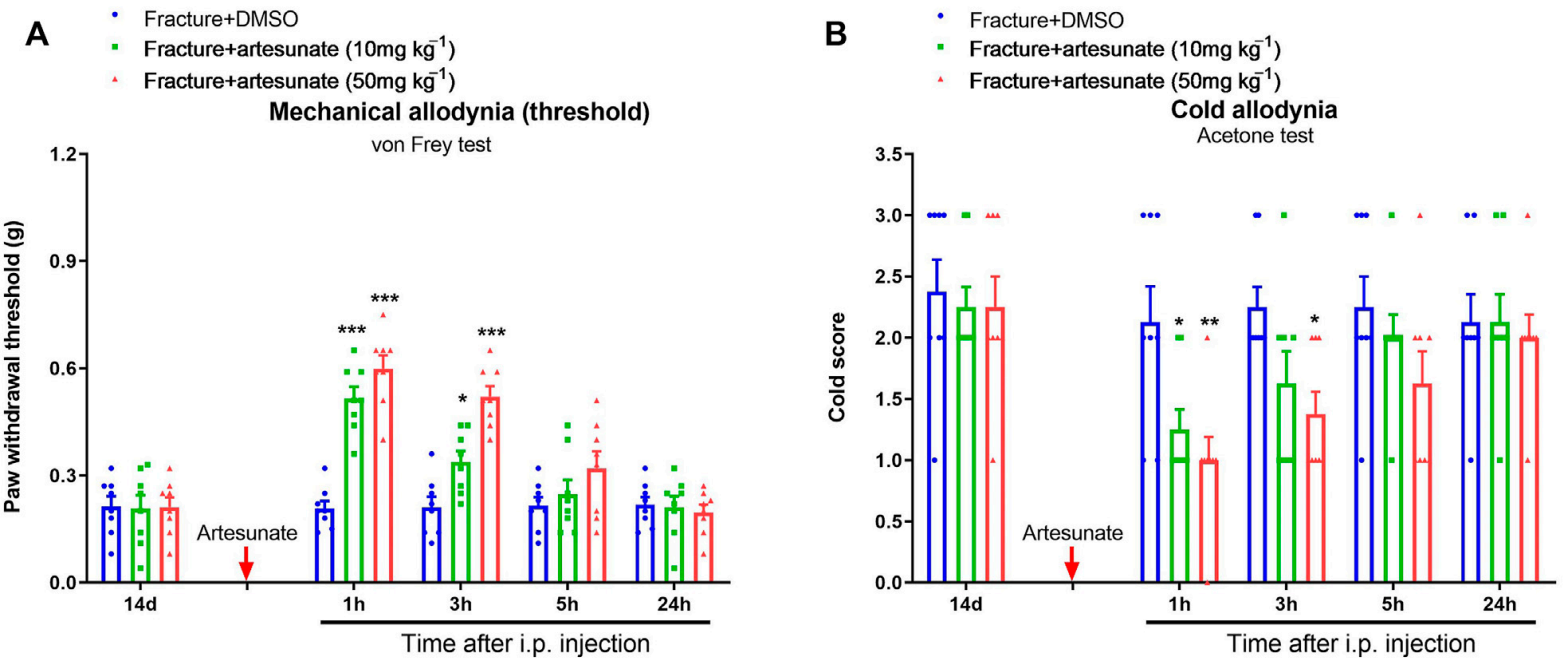
Systemic post-treatment of artesunate impairs the established chronic pain after tibial fracture and orthopedic surgery. Artesunate (10 and 50 mg kg^−1^, indicated by a red arrow) was intraperitoneally (i.p.) injected on day 14 (in the late phase) after tibial fracture with pin insertion. The development of mechanical allodynia was assessed by paw withdrawal mechanical threshold **(A)** in the von Frey test after tibial fracture. The development of cold allodynia was assessed by cold response scoring **(B)** in acetone test after tibial fracture. Results are mean ± SEM (*n* = 8) and analyzed by two-way ANOVA with Bonferroni *post hoc* comparisons. *p < 0.05, **p < 0.01, ***p < 0.001 vs. group fracture + DMSO.

Given that artesunate is often administered *via* intraperitoneal injection for the treatment of several diseases in rodents (Cheong et al., 2020; Bang et al., 2021), we finally investigated whether intraperitoneal artesunate therapy was also beneficial to chronic fracture pain. Thus, we administered artesunate (10 and 50 mg kg^−1^) following intraperitoneal injection on 14 days after orthopedic surgery. Interestingly, the systemic delivery of artesunate at 10 and 50 mg kg^−1^ relieved the fracture-induced mechanical allodynia and cold allodynia, as manifested by the elevation in paw withdrawal mechanical threshold [F (2, 105) = 31.23, *p* < 0.001, *n* = 8, two-way ANOVA; Figure 8A] and the decrease in cold response scores [F (2, 105) = 8.59, *p* < 0.001, *n* = 8, two-way ANOVA; Figure 8B]. Such analgesia of intraperitoneal artesunate sustained for 1–3 h.

## Discussion

In this current study, the major findings are as follows: first, tibial fracture originates from the persistent mechanical allodynia and cold allodynia with the spinal up-modulation of CCL21 and TREM2/DAP12 expressions following orthopedic surgery. Second, the intrathecal delivery of artesunate prevents fracture-associated allodynia in a dose-dependent manner and reduces fracture-caused CCL21 over-expression, as well as the TREM2/DAP12 accumulation in the spinal dorsal horn. Third, the postoperative therapy of both intrathecal and intraperitoneal artesunate attenuates the established fracture allodynia. Fourth, spinal CCL21 neutralization is sufficient to impair the generation and maintenance of fracture-associated pain. Fifth, the pre-administration of artesunate impairs exogenous CCL21-evoked acute inflammatory pain by reducing the spinal TREM2/DAP12 overload. Sixth, the pharmacologic inhibition of TREM2/DAP12 also ameliorates fracture-associated chronic pain and CCL21-elicited acute pain. These results therefore elucidate a previously undescribed role of artesunate as an alleviator of chronic pain following tibial fracture and orthopedic surgery by the inhibition of CCL21-dependent TREM2/DAP12 neuroinflammatory signaling.

Neuroinflammation driven by chemokines is a cardinal feature of chronic pain following peripheral tissue damage, nerve trauma, chemotherapy, and cancer (Ji et al., 2014; Ji et al., 2018; Baral et al., 2019). We previously identified the requirement for chemokine CCL1 and its receptor CCR8 in neuroinflammation and neuronal excitability in fracture-associated pain generation and chronification (Wang et al., 2020b). The contribution of caspase-6 to the upregulation of chemokine CCL21 in the development of opioid-induced hyperalgesia has been recently revealed (Wang et al., 2020a). Moreover, the spinal inhibition of caspase-6-dependent neuroinflammation is effective against functional potentiation in excitatory nociceptive synapses and pain chronification after tibial fracture (Cui et al., 2021). Given that caspase-6 regulates fracture-associated pain and CCL21 pathway is an important downstream effector of caspase-6 in spinal pain transmission (Wang et al., 2020a; Cui et al., 2021), we examined whether CCL21 contributes to persistent fracture pain. Herein, we initially reported the spinal long-lasting increase of CCL21 expression in mice with tibial fracture and orthopedic surgery, consistent with the time course of chronic mechanical allodynia and cold allodynia. This is the first study demonstrating that spinal CCL21 neutralization not only prevents but also reduces fracture-associated postoperative allodynia. Additionally, we revealed that exogeneous CCL21 intervention following spinal application elicits acute pain behaviors in animals. This evidence strongly suggests the identification of spinal CCL21 pathway in the development of persistent pain after fracture. Still, how spinal CCL21 mediates neuroinflammatory process in chronic fracture pain remains to be investigated.

It is noteworthy that the involvement of TREM2/DAP12 in neuron–microglia interactions is indispensable for neuroinflammation-associated pain perception in several rodent models (Guan et al., 2016; Kobayashi et al., 2016; Wang Y. et al., 2020). Especially, TREM2, as a pivotal factor for microgliosis, is upregulated and promotes the recruitment of cytokines in the pathogenesis of cisplatin-induced peripheral neuropathic pain (Ma et al., 2022). However, the requirement of TREM2 and DAP12 for the pathophysiology of chronic fracture pain is virtually unknown. Interestingly, this is the first study wherein spinal concentrations of TREM2 and DAP12 represent a robust elevation after tibial fracture with pin insertion and TREM2 neutralization impairs chronic fracture pain. Simultaneously, it is clarified, for the first time, that spinal exposure to CCL21 upregulates TREM2 and DAP12 levels and the pharmacological blockage of TREM2 reduces CCL21-caused acute pain. These detailed data point to the possibility that spinal CCL21 over-expression facilitates TREM2 and DAP12 accumulation to further cause nociception phenotypes and that inhibiting this may provide a novel therapeutic target for pain conditions.

Artemisinin and its derivatives perform a potent anti-neuroinflammatory protection on Alzheimer’s disease (Qiang et al., 2018), traumatic brain injuries (Zhou et al., 2020), and lipopolysaccharide (LPS)-induced cognitive dysfunction (Lin et al., 2021). Recent investigation has highlighted that artesunate therapy downregulates the severity of bacterial infection, the release of inflammatory mediators, nociception-like phenomena, and septic death (Bang et al., 2021). Artesunate is also effective against opioid-induced acute hyperalgesia and chemical irritant-induced acute inflammatory pain (Guruprasad et al., 2015; Zhang et al., 2022). However, no literature has mentioned the therapeutic role of artesunate in fracture-associated chronic pain. To the best of our knowledge, the present study is the first to uncover that repetitive injections of i.t. artesunate (10 and 100 μg but not 1 μg) prevent long-lasting mechanical and cold allodynia after tibial fracture and orthopedic surgery in a dose-dependent manner. We then discovered the mitigation of the established chronic fracture pain and the prevention of exogenous CCL21-induced acute inflammatory pain by the single application of artesunate (100 μg). Furthermore, this is the first study in which artesunate treatment disrupts spinal CCL21 over-expression and TREM2/DAP12 accumulation in fracture animals. Artesunate also reverses the exogenous CCL21-caused spinal overload of TREM2/DAP12. Strikingly, the intraperitoneal delivery of artesunate (10 and 50 mg kg^−1^) is sufficient and effective in abrogating fracture-associated chronic pain. Taken together, these results emphasize that artesunate therapy protects against the generation and maintenance of fracture-associated chronic pain through inhibiting spinal CCL21-dependent TREM2/DAP12 inflammatory signaling and microglia activation. Indeed, microgliosis is capable of raising excitatory neuronal responsiveness by promoting the secretion of tumor necrosis factor-α (TNF-α) (Berta et al., 2014). Thus, it will be of great interest to explore whether TNF-α is a potential downstream effector in the anti-nociceptive mechanisms of artesunate therapy.

Apart from its anti-inflammatory properties, artemisinin and its derivatives can activate the antioxidant system to exert potent neuroprotective effects on hydrogen peroxide (H_2_O_2_)-induced retinal neuronal dysfunction, sodium nitroprusside-induced cortical neurotoxicity, and neurodegenerative disease (Zheng et al., 2016; Yan et al., 2017; Li et al., 2019). Oxidative insult-related spinal nociception sensitization has been identified to be a cardinal step for the generation of fracture-induced pain (Guo et al., 2018; Guo et al., 2021). Accordingly, further investigations are warranted to study whether oxidative molecular signaling is involved in artesunate analgesia for fracture intervention. Previous reports disclosed that neuroinflammation underlies the central pain sensitization and allodynia initiation *via* α-amino-3-hydroxy-5-methyl-4-isoxazolepropionic acid (AMPA) receptor activation in dorsal horn neurons of fractured animals (Wang et al., 2020b; Cui et al., 2021). However, the connection between artesunate and AMPA receptor in our model remains largely undefined. There are several limitations to be considered. First, we did not evaluate the anti-nociceptive potency of artesunate treatment for females, which should be addressed by further studies. Second, given that all experiments in our study were performed using adult male C57BL/6J mice (8–10 weeks old) without significant different body weights, we selected the same dose of artesunate through intrathecal injection, but it is unclear whether this dose of artesunate is sufficient to animals with larger body weights. Third, the intrathecal injection of drugs or antibodies can both affect spinal cord and DRG, so it will be important to explore whether the CCL21-dependent TREM2/DAP12 pathway in DRG is involved in artesunate analgesia. Fourth, although we administered the spinal application of exogenous CCL21 protein and neutralizing antibody against CCL21 to elucidate the mechanism of artesunate analgesia, it will be of great importance to further utilize spinal knockdown and over-expression of CCL21 gene in future. Another limitation is the failure to investigate whether other artemisinin derivatives (such as dihydroartemisinin and artemether) are also effective in controlling chronic fracture pain.

In summary, the current findings recapitulate an unconventional pharmacological role of artesunate in the prevention and alleviation of fracture-associated postoperative pain by inhibiting spinal CCL21-dependent TREM2/DAP12 inflammatory processes. These results suggest that artesunate administration and CCL21 neutralization may generate innovative therapeutic concepts for a targeted neurotherapeutic strategy for fracture pain in clinics.

## Data Availability

The raw data supporting the conclusion of this article will be made available by the authors without undue reservation.

## Abbreviations

AMPA, α-amino-3-hydroxy-5-methyl-4-isoxazolepropionic acid; ANOVA, analysis of variance; CFA, complete Freund’s adjuvant; DAP12, DNAX-activating protein of 12 kDa; DMSO, dimethyl sulfoxide; H_2_O_2,_ hydrogen peroxide; i.p., intraperitoneal; i.t., intrathecal; LPS, lipopolysaccharide; SEM, standard error of mean; TNF-α, tumor necrosis factor-α; and TREM2, triggering receptor expressed on myeloid cells 2.
